# Trends in the Tissue Culture Techniques and the Synthesis of Bioactive Compounds in *Eurycoma longifolia* Jack—Current Status and Future Perspectives

**DOI:** 10.3390/plants13010107

**Published:** 2023-12-29

**Authors:** Sani Sale, Sreeramanan Subramaniam, Mohamad Fadhli Mad’ Atari

**Affiliations:** 1 School of Biological Sciences, Universiti Sains Malaysia (USM), Georgetown 11800, Penang, Malaysia; 2 Department of Botany, Gombe State University, P.M.B 127, Gombe 760214, Nigeria; 3 Centre for Chemical Biology (CCB), Universiti Sains Malaysia (USM), Bayan Lepas 11900, Penang, Malaysia; 4 Department of Biology, Faculty of Science and Technology Universitas Airlangga, Surabaya 60115, Indonesia

**Keywords:** *Eurycoma longifolia*, plant tissue culture, elicitation, biosynthesis, bioactive compounds

## Abstract

Over the last two decades, there has been a concerted effort by researchers to mass propagate *Eurycoma longifolia* and improve the yield of its very important and sought-after anti-cancer and aphrodisiac bioactive compounds. To achieve this, various techniques have been used to mass propagate and improve the yield of these bioactive compounds in tissue cultures. These techniques include the optimization of media conditions and application of various types and combinations of plant growth regulators (PGRs). In addition, some elicitation techniques have been used to improve the synthesis of these bioactive compounds. However, in comparison with other herbal species with similar economic importance, many techniques have not been applied to *E. longifolia*. Adopting the most recent methodologies would ensure efficiency and sustainability in the in vitro production of bioactive compounds in *E. longifolia*. Therefore, in this review, we present an up-to-date record on the success stories in the tissue culture techniques and synthesis of bioactive compounds. In addition, we attempted to identify some of the missing links on the road to the effective and sustainable biotechnological utilization of this super important biological resource.

## 1. Introduction

*Eurycoma longifolia* Jack is a versatile tree of the Simaroubaceae family, popularly known as the Quassia family. It belongs to the genus *Eurycoma* together with two other two species, namely, *Eurycoma apiculata* A. W. Benn., which occurs in Malaysia and Indonesia, and *E. harmandiana* Pierre, which grows along the axis between Thailand and Laos. Although these species share some similarities in terms of chromosome number [1] and basic phytochemicals [2], *E. longifolia* is more widespread and more popular, thanks to its numerous bioactive compounds [2] and uses. It is native to southeast Asia, occurring mainly in Indonesia, Malaysia, Singapore, and Thailand. It is also found in Brunei Darussalam, Cambodia, Laos, Vietnam, southern Myanmar and the Philippines [3,4,5]. *E. longifolia* has various local names such as Tongkat Ali in Malaysia, Pasak Bumi in Indonesia, Ian-don in Thailand, Cây bách bệnh in Vietnam, and Tho nan in Laos [6].

*E. longifolia* is a medium-sized tree that typically reaches a height of about 15 to 18 m as undergrowth in forests [7]. This wild natural resource served as the sole source of tongkat ali products until recently, when growing demand exerted pressure on the wild resource, leading to the establishment of commercial plantations [8].

*E. longifolia* is regarded as one of the most valuable medicinal plants. In Vietnam, it is listed in the pharmacopoeia, and it is locally known as “chy ba binh”, which literally means tree that cures hundreds of diseases [9]. It is considered a national treasure in Malaysia [10] and a popular medicinal plant in Indonesia [11]. *E. longifolia* is utilized in both traditional herbal medicine and modern pharmaceutical applications. Additionally, it is employed in food supplements and the cosmetic industry. The multitude of applications has contributed to elevating *E. longifolia*’s status in both the commercial and scientific realms.

Traditionally, various parts of the *E. longifolia* tree are used to treat many diseases, which include skin itching, dysentery, stomach worms, diarrhea, and fever [12]. The root extract of *E. longifolia* is highly valued for its aphrodisiac properties and its efficacy in treating chronic diseases such as cancer and diabetes [13]. It is also employed for conditions such as leukemia, syphilis, fever, and osteoporosis. Additionally, it serves as an antibiotic, aids in slowing the aging process, and helps reduce stress and anxiety. Moreover, it is used in addressing gynecological disorders [14].

Scientific experiments using in vitro systems, animal models, and clinical trials have established the antimalarial [15], cytotoxic, anticancer [16,17,18,19], antidiabetic, aphrodisiac, proandrogenic, and antimicrobial effects [20,21,22] of *E. longifolia*. In addition, its efficacy in treating male sexual dysfunctions [23] and osteoporosis [24] has been confirmed. Furthermore, clinical studies have shown that eurycomanone, the major bioactive molecule in *E. longifolia*, is effective against lung, breast, gastric, and colon cancer [25]. It also exhibits wound healing properties [26] and effectiveness against bacteria, fungi, protozoa [27,28], dengue [29], and coronavirus [30].

*E. longifolia* has been extensively researched for its phytochemicals and bioactive compounds isolated from its root, leaf, and stem [31]. These compounds form a group of quassinoids/degraded triterpenoids [32], including eurycomalactone, eurycomanone, eurycomanol, and others [33,34,35,36]. Abubakar et al. [37] reported over 70 bioactive compounds from various parts of *E. longifolia* in their review. In the last decade, numerous new bioactive compounds have been reported [29,32,38,39,40,41,42,43,44]. This trend is expected to continue.

The products of *E. longifolia* are gaining public acceptance across the globe, with hundreds of products being registered by the relevant authorities. The Malaysian Ministry of Health estimated the total value of *E. longifolia* at USD 1.7 billion, projecting an annual increase of 15% [45]. This lucrative market has attracted significant interest, raising concerns about product adulteration [46] and emphasizing the need for product authentication [47,48,49].

The approved products are already in international markets [50], leading to increased pressure on resources and prompting the implementation of government laws to ensure sustainability [45]. Ensuring sustainability in the utilization of plant resources can be achieved through effective propagation techniques. In the case of *E. longifolia*, numerous research reports focus on in vitro propagation and various strategies for enhancing the synthesis of bioactive compounds.

**Figure 1:**
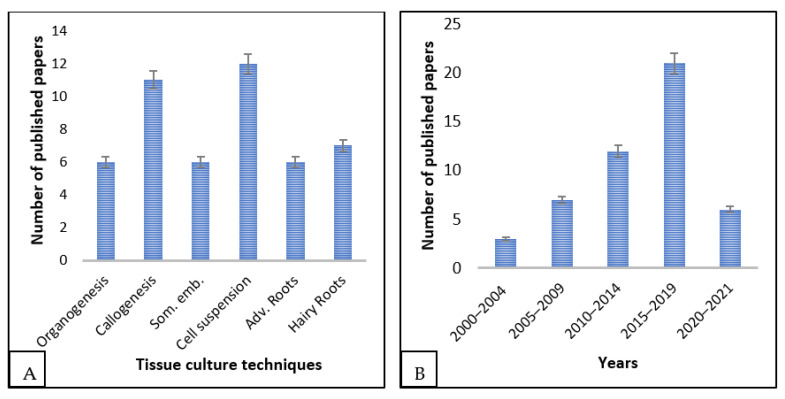
Current trend in *E. longifolia* tissue culture; (**A**) various tissue culture techniques employed in *E. longifolia*, (**B**) occurrence of publications of *E. longifolia* over the years. The last bar represents only two years.

The first report on *E. longifolia* tissue culture surfaced in 2000. Since then, numerous papers, covering various tissue culture techniques, have been published (Figure 1A). Notably, from 2015 to 2019, there has been a surge in interest in the tissue culture of this medicinal tree (Figure 1B), which is expected to intensify in the coming years. 

**Figure 2:**
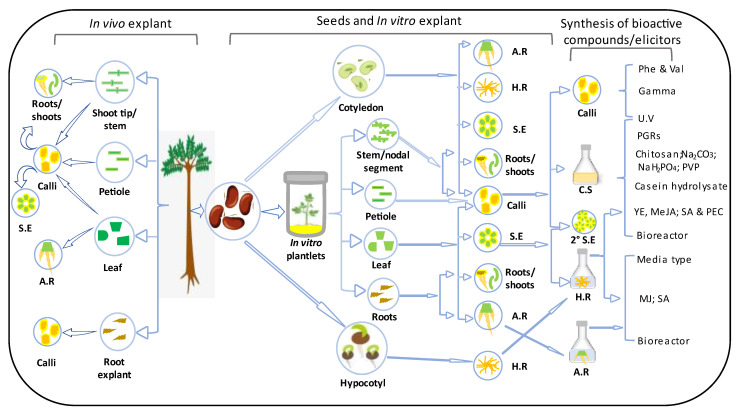
Summary of the successes in the tissue culture and in vitro synthesis of bioactive compounds in *E. longifolia*. S.E = somatic embryo; A.R = adventitious roots; H.R = hairy roots; C.S = cell suspension; Phe = phenylalanine; Val = valine; PEC = pectin; YE = yeast extract; MeJa = methyl jasmonate; JA = jasmonic acids; SA = salicylic acid; 2° = secondary; Na_2_CO_3_ = sodium carbonate; NaH_2_PO_4_ = monosodium phosphate; PVP = polyvinylpyrrolidone.

Therefore, there is a need to compile information for convenient access, facilitating the comparison of techniques and aiding decision-making. This report serves as a comprehensive guide for the development and utilization of *E. longifolia* resources, offering updates on successful tissue culture techniques and in vitro synthesis of bioactive compounds (Figure 2). Recommendations are also provided for future possibilities to ensure effective and sustainable biotechnological exploitation.

## 2. Tissue Culture Techniques for Mass Propagation in *E. longifolia*

### 2.1. Techniques for Direct Organogenesis

The induction of organs, such as roots and shoots, directly on the explant is a micropropagation technique that allows for the swift clonal mass propagation of plants. In *E. longifolia*, various plant growth regulators (PGRs) have been utilized to stimulate direct organogenesis (Table 1). Hussein et al. [51], using shoot tips as explants, induced shoots on Murashige and Skoog (MS) medium with 5.0 mg/L kinetin and roots on 0.5 mg/L indole-3-butyric acid (IBA). In a similar vein, Hussein et al. [52], utilizing roots as explants, generated shoots on Driver and Kuniyuki Walnut (DKW) media with 1.0 mg/L zeatin, and induced roots on MS medium with 0.5 mg/L IBA. Similarly, stems were used to induce shoots on woody plant medium (WPM) supplemented with 2.0 mg/L each of 6-benzylaminopurine (BAP) and zeatin, and roots were induced on MS medium with 0.5 mg/L IBA. Using nodal segments, cotyledons, and in vitro leaves as explants, shoots were induced on both half-strength and full-strength MS supplemented with 0.5 mg/L BAP and 1.0 mg/L BAP, respectively. Additionally, roots were induced on half-strength MS supplemented with 10 mg/L and 0.5 mg/L IBA and full-strength MS supplemented with 0.5 mg/L IBA [53,54,55]. The light conditions used in the above studies were 16L: 8D with an intensity of 35–150 µmol/m^2^/s. 

From the above results, it can be deduced that BAP (0.5–2.0 mg/L) resulted in a higher percentage and larger shoot formation regardless of the explants used. Similarly, IBA at 0.5 mg/L was found to be best for root induction compared to other auxins. This shows that these PGRs are superior to others in the organogenesis of *E. longifolia*.

### 2.2. Techniques for Callogenesis and Callus Elicitation

Callus induction is a crucial event in various tissue and cell culture systems, as it can function as a transient tissue for organogenesis, somatic embryogenesis, cell culture [56], and more. Additionally, it can serve as a latent tissue for the synthesis of bioactive compounds. In Table 2, the main techniques for the induction, proliferation, and elicitation of callus in *E. longifolia* are summarized. Siregar et al. [57] tested the effects of different genotypes, media, and naphthaleneacetic acid (NAA) concentrations on callus induction using leaves as explants. The result showed that MS modification and the genotype have effects on callus induction.

Siregar et al. [58] investigated the effects of BAP and NAA on callus formation using various explants. They found that MS supplemented with 8.0 mg/L NAA and 2.0 mg/L BAP yielded the highest callus biomass on petioles. In contrast, Mahmood et al. [59], using various plant parts as explants, discovered that different concentrations of 2,4-dichlorophenoxyacetic acid (2,4-D) and picloram were effective in inducing callus. Similar results have shown that 1.0 mg/L 2,4-D is effective in callus induction [60].

Studies on the effects of different concentrations of 2,4-D and NAA revealed that 1.0 mg/L NAA plus 1.0 mg/L BAP induced callus on leaves, while 1.0 mg/L 2,4-D plus 1.0 mg/L BAP induced callus on petioles [61]. Furthermore, the subculture of callus on 1.5 mg/L NAA and 1.0 mg/L kinetin resulted in improved biomass [62].

The production of bioactive compounds in *E. longifolia* callus has been documented. According to Rosli et al. [63], higher levels of methoxycanthin-6-one were reported on quarter-strength MS (¼ MS) with 2% fructose and 2 mg/L dicamba, along with the addition of 1.65 × 10^−2^ mg/L phenylalanine. Furthermore, gamma irradiation [64] has been shown to reduce callus biomass, total phenolics, and flavonoids. Interestingly, increasing the dose to around 60 Gy enhanced the synthesis of soluble protein. This suggests that gamma irradiation at a specific dose can stimulate changes in certain metabolic pathways. However, the available data are insufficient to fully explain the mechanisms involved.

**Table 2:** Summary of the techniques for callogenesis and callus elicitation in *E. longifolia*, indicating the explant, the media, and the culture conditions used with the results.

Explants	Media + PGR + Additives	Other Culture Conditions	Morphogenic Response/Outcome	Refs.
Leaves	MS + NAA and various macro nutrients	Various plant sources	Eu 9 plant, pH 5.75, and modified MS formed more callus	[57]
Leaves, stems, and petioles	MS + BAP, NAA	24L: 0D lighting of 30 µmol/m^2^/s	8.0 mg/L (43.01 µM) NAA + 2.0 mg/L (8.88 µM) BAP formed higher callus on petioles, while 10 mg/L NAA formed callus on leaves	[58]
All plant parts	MS, SH, WH, and B5 + auxins, sugars, and amino acids		¼ MS + 2% fructose + 2 mg/L dicamba and 1.65 × 10^−2^ mg/L phenylalanine produced higher 9-MCO	[63]
All plant parts	MS + 2,4-D, dicamba, picloram, NAA, and IAA	Continuous dark	1.0–4.0 mg/L 2,4-D produced callus on leaf, petioles, rachis, stem, root, and cotyledon explants, etc.	[65]
Callus	MS + 1 mg/L 2,4-D	16L: 8D lighting of 15 µmol/m^2^/s and gamma	Gamma radiation decreased biomass, total phenol, and flavonoids but improved soluble protein at 60 Gy	[64]
Leaves and petioles	MS + 2,4-D, NAA, BAP, and KIN		1.0 mg/L (1.0 ppm) NAA + 1 mg/L BAP induced callus on leaves, and 1.0 mg/L (1.0 ppm) 2,4-D + 1 mg/L BAP induced callus on petioles	[61]
Root segments	MS + 1 mg/L 2,4-D	16L: 8D lighting of 40 µmol/m^2^/s	Treatment produced callus	[60]
Callus	MS + 2,4-D, NAA, and KIN	8L: 16D lighting	1.5 mg/L NAA and 1.0 mg/L KIN produced better biomass	[62]

Key: PGRs = plant growth regulators; BAP = 6-benzylaminopurine; KIN = kinetin; NAA = 1-naphthaleneacetic acid; 2,4-D = 2,4-dichlorophenoxyacetic acid; IAA = indole-3-acetic acid; MS = Murashige and Skoog medium; SH = Schenk and Hildebrandt medium; WH = White’s medium; B5 = Gamborg (B5) medium; Gy = Gray; 9-MCO = 9-methoxycanthin-6-one; Eu 9 = code given by the author.

Different photoperiods, including continuous dark and different light intensities ranging from 15 to 50 µmol/m^2^/s [58,65], were applied. The multiplicity in induction conditions highlights that callus in *E. longifolia* can be induced from different explants using various types, combinations, and concentrations of PGRs and under different light conditions. However, leaves seem to be the predominant explants used, with 2,4-D and NAA emerging as the most frequently employed PGRs.

### 2.3. Techniques for Induction and Multiplication of Somatic Embryos (SEs)

Somatic embryogenesis is a significant event that can enhance the success of clonal and mass propagation, cryopreservation, synthetic seed production, and/or genetic improvement of important plant species [66,67,68,69,70]. In *E. longifolia*, the successful induction of somatic embryos has been reported (Table 3).

Aziz et al. [71] induced direct somatic embryos (SEs) and indirect ones (via embryonic calli (EC)) from immature cotyledons on NAA and 2,4-D, respectively. Similarly, Hussein et al. [72], using different explants, concluded that cotyledonary tissues produced EC on 1.0 mg/L 2,4-D, and EC subculture on 0.5 mg/L kinetin plus 1 mg/L 2,4-D resulted in a higher yield. Furthermore, EC were used to induce SEs on liquid media containing 1–2.5 mg/L 2,4-D, 2.0 mg/L BAP, and kinetin [73]. Additionally, Dalila et al. [74] found that the highest percentage of SE was produced using modified MS containing 0.1 mg/L zeatin, 0.2 mg/L IBA, and 0.12 mg/L TDZ. Mohd et al. [75] also utilized the mentioned liquid media and varied the immersion frequencies in RITA^®^ bioreactors. They found that an immersion frequency of 5 min every 4 h produced the highest SE.

From all the studies above, it is evident that cotyledons are the only explants to successfully produce SEs on MS supplemented with NAA, 2,4-D, and zeatin. This may result from specific cells in cotyledons that are more responsive to these hormones, leading to the initiation of embryonic cells. Furthermore, SEs can be induced in *E. longifolia* both in total darkness and different photoperiods, demonstrating the light independency of the process that leads to somatic embryo formation.

## 3. Techniques for Improving the Synthesis of Bioactive Compounds in *E. longifolia*

### 3.1. Techniques for the Establishment of Cell Suspension Culture and Synthesis of Bioactive Compounds

Plant cell suspension cultures are increasingly being utilized for synthesizing bioactive compounds. This approach, applied to *E. longifolia*, offers a convenient method for producing compounds for agricultural, pharmaceutical, and industrial applications. Various culture conditions and elicitation techniques have been studied (as summarized in Table 4). It should be noted that the initial steps of callus formation have already been presented in the earlier sections of this review. Therefore, only the steps involved in the establishment of cell suspensions from the callus are highlighted in this section.

One of the earliest reports is that of Siregar et al. [57], in which the effects of adjusting MS nutrients and pH level on growth were tested. The best results were recorded at pH 5.75 in the modified MS. Similarly, under these conditions, variations between different cell sources were tested [76]. The results indicated that different cell lines react differently, with Eu9 producing the highest biomass and Eu8 producing the highest level of alkaloids. Other studies revealed that MS supplemented with 0.5 mg/L (2.69 µM) NAA and 0.25 mg/L (1.13 µM) 2,4-D produced more 9-methoxycanthin-6-one and 9-hydroxycanthin-6-one alkaloids [58], and MS with modified nutrients yielded even better levels of biomass and alkaloids [77].

Studies on the effects of carbon and nitrogen sources have shown that glucose and potassium nitrate (KNO_3_) produced the highest cell growth and soluble protein in CS cultures [78]. In a similar study, Shim et al. [60] revealed that full-strength MS supplemented with 3.0 mg/L NAA, 3% sucrose, and 0:60 ratio of NH_4_^+^:NO_3_^−^ produced better biomass. Additionally, the content of eurycomanone in the CS improved on MS plus 1.2 mg/L NAA and 1.0 mg/L kinetin [79].

**Table 4:** Summary of various techniques for establishment and elicitation of cell suspension culture in *E. longifolia* including the media and culture conditions and the corresponding outcome.

Medium + PGR	Suspension Culture Conditions	Objective	Outcome	Refs.
MS + NAA	1.0 g cells shaken in 20 mL MS at 130 rpm under 24L: 0D lighting	Effects of pH and Macro-nutrients	Cells grew better in 5.75 pH and modified MS	[57]
MS + NAA	1.0 g cells shaken in 20 mL MS at 130 rpm under 24L: 0D lighting	Effects of cell source and pH on canthin-6-one	Eu9 produced highest biomass while Eu8 produced more canthin-6-one at pH 5.75	[76]
MS + NAA and 2,4-D	0.5 g cells shaken in 20 mL MS at 130 rpm	Effects of PGR on growth and synthesis	MS + 0.5 mg/L (2.69 µM) NAA and 0.25 mg/L (1.13 µM) 2,4-D gave better 9-MCO and 9-HCO alkaloid	[58]
MS + 0.5 mg/L NAA and 0.25 mg/L 2,4-D	1.0 g cells shaken in 20 mL MS at 130 rpm under 18 μE/m^2^/s	Effects of modified MS	Modified MS formed more biomass and biosynthesis	[77]
Modified MS	Callus in MS + Chitosan, Na_2_CO_3_, NaH_2_PO_4,_ and PVP and 24L: 0D lighting of 32.5 µmol/m^2^/s	Elicitation	100 g/L chitosan produced more biomass; 150 produced the highest 9-MCO and 9-HCO; 2.0 mg/L and 20 mg/L NaH_2_PO_4_ produced the highest biomass and alkaloid, respectively	[80]
MS + 0.5 mg/L NAA and 0.25 mg/L 2,4-D	1.0 g cells in 25 mL MS + casein hydrolysate and shaking at 130 rpm under 1525 lux of light	Effects of casein hydrolysate and light on biomass and 9-MCO	0.1–2.0% casein hydrolysate and 1525 lux of light improved the synthesis of 9-MCO	[81]
MS + 1.0 mg/L 2,4-D; sugar and nitrogen sources	Callus shaken at 100 rpm under 16L: 8D lighting	Optimization for growth	Glucose and KNO_3_ produced the highest cell growth, soluble protein, and activity of peroxidase	[78]
MS + 2,4-D and KIN	Cells suspended in ½ MS and shake at 100 rpm	Elicitation with UV	UV + 1.1 mg/L (1.1 ppm) 2,4-D and 1.0 mg/L (1.0 ppm) KIN improved alkaloids synthesis	[82]
MS + IBA, NAA, sugar, and nitrogen sources	5.0 g cells shaken in 100 mL MS at 110 rpm under 40 µmol/m^2^/s, 16L: 8D lighting	Optimization for growth	Full-strength MS, 3.0 mg/L NAA, 3% sucrose, and 0:60 NH_4_^+^:NO_3_^−^ produced better biomass	[60]
MS + NAA and KIN; sugar sources	3.0 g callus shaken in 50 mL MS at 120 rpm under 500 lux	Optimization for eurycomanone synthesis	1.2 mg/L NAA and 1.0 mg/L KIN produced more eurycomanone	[79]
MS + NAA and KIN; YE, MeJa, and SA	3.0 g callus shaken in 50 mL MS at 120 rpm under 500 lux	Elicitation	200 mg/L YE, 20 µM each of MeJa and SA produced more eurycomanone	[83]
MS + NAA and 2,4-D; YE, PEC, and VAL	1.0 g callus shaken in 20 mL MS at 120 rpm under 32.5 µmol/m^2^/s	Biotic elicitation	Elicitation improved biosynthesis	[84]

Key: PGRs = plant growth regulators; KIN = kinetin; IBA = indole-3-butyric acid; NAA = 1-naphthaleneacetic acid; 2,4-D = 2,4-dichlorophenoxyacetic acid; MS = Murashige and Skoog medium; YE = Yeast extract; MeJa = methyl jasmonate; SA = salicylic acid; PEC = pectin; VAL = valine; PVP = polyvinylpyrrolidone; 9-MCO = 9-methoxycanthin-6-one; 9-HCO = 9-hydroxycanthin-6-one; Eu 8 and Eu 9 = code given by the author.

Different elicitation techniques using both biotic and abiotic factors on the CS of *E. longifolia* have also been reported. Keng et al. [80], using modified MS and different elicitation agents, reported that 100–150 g/L chitosan produced higher biomass and 9-methoxycanthin-6-one and 9-hydroxycanthin-6-one. However, NaCO_3_ and PVP inhibited growth but had no effect on alkaloid synthesis. Similarly, Siregar et al. [81] tested the elicitation effects of casein hydrolysate and found that 0.1–2% casein hydrolysate, along with 1525 lux of light, improved the synthesis of 9-methoxycanthin-6-one. Additionally, the effect of UV irradiation on the CS resulted in improved the alkaloids content [82].

In addition to physical and chemical elicitation, biotic elicitors such as yeast extract (YE), methyl jasmonate (MeJa), salicylic acid (SA), pectin (PEC), and valine (VAL) were tested in *E. longifolia*. Nhan and Loc [83] found that 200 mg/L YE and 20 µM each of MeJa and SA were ideal for enhancing the synthesis of eurycomanone. Similarly, Kwan et al. [84] reported that YE, PEC, and VAL have positive effects on the synthesis of bioactive compounds in *E. longifolia*.

From Table 4, it can be inferred that the most appropriate PGR combination for establishing cell suspension cultures in *E. longifolia* is either a combination of 5.0 mg/L NAA and 2.5 mg/L 2,4-D or 1.2 mg/L NAA and 1.0 mg/L kinetin. This shows the cells can respond positively to a variety of PGRs. Additionally, the optimal culture density and shaking frequency were determined to be 1.0 g/20 mL and 120 rpm, respectively. Biotic elicitation techniques have been shown to be efficient in enhancing the cell biomass and synthesis of bioactive compounds. This could help positively towards the mass production of phytochemicals in *E. longifolia*. 

### 3.2. Induction, Proliferation, and Elicitation of Adventitious Roots (ARs)

Adventitious roots (ARs) are induced to propagate true-to-type plants and/or to produce bioactive compounds from medicinal plants. In *E. longifolia*, there are only a few reports on the induction and/or elicitation of ARs (Table 5).

Ali et al. [85] observed that ARs could be produced on leaf explants by adjusting the MS medium to contain half the normal quantity of nitrate plus 5.0 mg/L IBA. Similarly, Lulu et al. [86] achieved successful ARs production on leaf explants using ¾ MS strength and 3.0 mg/L IBA. Consistent results were reported by Cui et al. [87], where ¾ MS with 3.0 mg/L IBA led to improved ARs and metabolite production. Giap et al. [88], using cotyledons as explants and testing various PGR combinations, established that MS with 1.5 mg/L NAA and 0.1 mg/L BA is optimal for ARs induction. Furthermore, 3.0 mg/L NAA with 50 g/L sucrose was found to induce better ARs, while elicitation with MeJa showed a reduction in alkaloid contents [89].

**Table 5:** Summary of the techniques for induction, growth, and elicitation of adventitious roots (ARs) showing the explants, media, and culture conditions and the corresponding outcome.

Explants	Media + PGR and Other Culture Conditions	Morphogenic Response/Outcome	Refs.
Leaves	MS + IBA, IAA, NAA, and sucrose	MS (½ nitrate) + 5.0 mg/L IBA produced better ARs	[85]
Leaves	MS + IBA, NAA, and IAA; different carbon source in continuous dark	MS + 3.0 mg/L NAA and 50 g/L sucrose produced better biomass	[89]
In vitro roots	MS + 5.0 KIN and MeJa under 16L: 8D lighting of 150 µmol/m^2^/s	MeJa reduced alkaloids concentration	[90]
In vitro leaves	¾ MS + 3.0 mg/L IBA + 30 g/L sucrose in dark condition	Treatment produced ARs	[86]
In vitro cotyledons	MS + BA and NAA under 16L: 8D lighting of 2500–3000 lux	1.5 mg/L NAA and 0.1 mg/L BA produced ARs on cotyledons	[88]
Leaves	Diff. media type, and IBA and sucrose in dark condition	¾ MS + 3.0 mg/L IBA and 30 g/L sucrose produced better ARs and metabolites	[87]

Key: PGRs= plant growth regulators; BA = benzyl adenine; KIN = kinetin; IBA = indole-3-butyric acid; NAA = 1-naphthaleneacetic acid; IAA = indole-3-acetic acid; MS = Murashige and Skoog medium; MeJa = methyl jasmonate.

From these results, it can be deduced that ARs are mostly induced using leaves as explants with 3.0–5.0 mg/L IBA under dark conditions. However, other PGR combinations, such as NAA and BA, are suitable for cotyledonary explants. This explains the facts that different tissues response differently to different PGRs depending on their cell types, endogenous hormones, and total physiology.

### 3.3. Transformation with A. rhizogene and Induction of Hairy Roots (HRs)

HRs are engineered to serve as living factories for producing valuable phytochemicals for pharmaceutical, cosmetic, and agricultural applications. Inducing HRs marks a milestone in plant biotechnology, enabling the large-scale production of bioactive compounds without the need for PGRs [91,92,93,94].

In *E. longifolia*, limited reports document the successful generation of HRs (Table 6). Balakrishnan et al. [95] achieved the successful generation of HRs using somatic embryos (SEs) with two strains of *A*. *rhizogenes* (AR12 and AR14). The infected tissues were cultured in the dark for three days, and ultimately, a transient expression of the β-glucuronidase (GUS) gene was detected.

In research conducted by Danial et al. [96], various explants and strains of *A*. *rhizogenes* were tested, revealing that two strains induced HRs on the plants’ hypocotyl region. Similarly, Ngoc et al. [39] successfully produced HRs from in vitro cotyledons and hypocotyls using *A*. *rhizogenes* strain ATTC 15834. 

The generation of hairy roots from *E. longifolia* may not have received significant attention or could have presented challenges. Nevertheless, the current results suggest that hairy roots can be induced from hypocotyl and cotyledon tissues, which are somewhat related in plants, explaining their ability to differentiate into HRs after successful transformation. Furthermore, successful transfection can be achieved through co-culturing tissues and bacteria using various media compositions. 

### 3.4. Improving the Synthesis of Bioactive Compounds in Hairy Roots (HRs) Culture

Following the successful induction of HRs, the focus shifts to optimizing the growth conditions and synthesizing target bioactive compounds. HRs exhibit rapid growth even without PGRs, prompting the exploration of factors such as media type, culture density, and external elicitors. The effects of different media and the elicitation on *E. longifolia* HRs are summarized in Table 7. 

Studies investigating the effects of media types and elicitation techniques on the growth and synthesis of hairy roots revealed that Gamborg B5, Schenk and Hildebrandt media (SH), and woody plant medium (WPM) supported the maximum growth and synthesis of alkaloids [97,98]. Furthermore, elicitation with jasmonic acid (JA), YE, MeJa, and SA enhanced the synthesis of canthin-6-one alkaloids [98,99].

**Table 7:** Techniques of maintenance and elicitation of HR cultures in *E. longifolia* indicating various media types and their effects.

Medium + Additives	Culture Conditions	Objective	Outcome	Refs.
B5, ½ B5, SH, ½ SH, N6, and ½ N6	0.2 g HRs agitated at 110 rpm	Effects of media type	B5 supported maximum growth and production of alkaloid	[97]
MS + MeJa + SA	0.2 g HRs in 50 mL MS at 110 rpm	Elicitation for 9-MCO production	MeJa and SA at 0.1 mM each produced high amounts of 9-MCO	[99]
WPM, MS and SH + JA and YE	0.3 g HRs in 100 mL media and agitated at 80 rpm	Elicitation for improved biomass and 9-MCO	SH and WPM produced the best biomass and 9-MCO alkaloids, and JA and YE elicitation improved only 9-MCO synthesis	[98]

Key: B5 = Gamborg B5 medium; SH = Schenk and Hildebrandt media; N6 = Chu (N6) medium; MS = Murashige and Skoog medium; WPM = woody plant medium; MeJa = methyl jasmonate; JA = jasmonic acid; YE = yeast extract; SA = salicylic acid; 9-MCO = 9-methoxycanthin-6-one.

Based on the results presented in Table 7, it can be concluded that elicitation techniques have positive effects on the synthesis of bioactive compounds in the HRs of *E. longifolia*. This can therefore add to the inherent abilities of HRs. The findings also indicate that HRs can grow well on a variety of media. Furthermore, agitation at 110 rpm in dark conditions is the most frequently employed technique.

### 3.5. Upscale Production of Bioactive Compounds in Bioreactors

Bioreactors are utilized for the scaled-up production or synthesis of bioactive compounds in living cells or tissues. Bioreactors offer a superior alternative to other culture systems [100] because of their efficient contact between cells or tissues and the medium, as well as their improved aeration and growth.

There are only a few reports on the use of bioreactors for the large-scale production of bioactive compounds in *E. longifolia* (Table 8). Natanael et al. [82] tested the effects of bioreactors and UV irradiation on CS. The results showed that UV has a positive effect on the synthesis of canthin-6-one and β-carboline. In a similar study, Shim et al. [60] found out that a CS density of 50 g/L at an aeration rate of 0.3 vvm was optimal for growth and the synthesis of phenolics.

In other studies, adventitious roots (ARs) were fed to a bioreactor at a density of 5.0 g/L and an aeration rate of 0.1 vvm to test the effects of nitrogen sources [86]. The results showed that a ratio of 1:2 NH_4_^+^ and NO_3_^−^ was optimal for growth and synthesis. In Fan et al. [101], ARs were fed to bioreactors containing ¾ MS to optimize the growth conditions. The results showed that 40 g/L sucrose, a 5.0 g/L cell density, and a 0.05 vvm aeration rate were optimum for the growth synthesis of eurycomanone. Additionally, the effects of bioreactors on the HRs resulted in improved biomass and synthesis of canthin-6-one alkaloids compared to shake flasks [102].

In summary, three types of tissues (CS, HRs, and ARs) have been reported for use in bioreactors. These tissues exhibit little variation in their culture conditions. Generally, different types and strengths of media are applicable. ARs and HRs were cultured in the dark, while CS requires light. The aeration rate ranged between 0.05 and 0.3 vvm. Therefore, the large-scale production of *E. longifolia* products is possible using bioreactors.

**Table 8:** Techniques for the large-scale production of bioactive metabolites in *E. longifolia* using bioreactor including the explant used, the culture condition, and the results.

Explants	Bioreactor Type	Culture Media/Condition	Inoculation Condition	Outcome/Opt. Condition	Refs.
CS	Bubble column	½ MS with 25 g/L sucrose + 1.1 and 1.0 mg/L 2,4-D and KIN	5.0 g/L cells at 0.3 vvm aeration and 18L: 6D UV	UV improved canthin-6-one and β-carboline	[82]
ARs	5 L balloon- type bubble	¾ MS + IBA and NAA and varying ratios of NH_4_^+^:NO_3_^−^	5.0 g/L ARs at 0.1 vvm aeration in dark	IBA, NAA, and 1:2 NH_4_^+^:NO_3_^−^ are optimum	[86]
CS	5 L balloon-type bubble	MS + 3.0 mg/L NAA, 3% sucrose and 0:60 NH_4_^+^:NO_3_^−^	40–80 g/L cell at 0.05–0.3 vvm and 16L: 8D lighting of 40 µmol/m^2^/s	50 g/L and 0.3 vvm improved biomass and phenols	[60]
HRs	20 L spherical bubble	Liq. WPM with 30 g/L sucrose and 40 mg/L YE	3.0 g/L HRs inoculated at 1.5 vvm in dark	Bioreactor improved biomass and synthesis of canthin-6-one alkaloids	[102]
ARs	5 L bubble column	¾ MS with diff. sucrose cons. + 3 mg/L IBA	2.5–5.0 g/L ARs inoculated at 0.05–0.1 vvm in dark	40 g/L sucrose, 5.0 g/L density, and 0.05 vvm were optimum for biomass and eurycomanone synthesis	[101]
HRs	5 L bioreactor	MS basal medium	Dark conditions	Biomass improved by 20-fold	[103]

Key: CS = cell suspension; ARs = adventitious roots; HRs = hairy roots; KIN = kinetin; IBA = indole-3-butyric acid; NAA = 1-naphthaleneacetic acid; MS = Murashige and Skoog medium; WPM = woody plant medium; YE = yeast extract.

## 4. Future Perspectives

Despite considerable efforts to enhance the yield of bioactive metabolites in *E. longifolia*, there is still a need for improvement to ensure sustainability. Adopting recent techniques already applied to other valuable medicinal plants would further enhance the synthesis of metabolites. The following sections briefly outline promising areas in tissue culture and biotechnological advancements that have not yet been applied to *E. longifolia*.

### 4.1. Elicitation Techniques

Elicitation induces stress in cells and tissues, enhancing secondary metabolite production. Biotic elicitation agents (bacteria, fungi, etc.) and abiotic stressors (heavy metal ions, UV radiation, nanoparticles, etc.) are utilized in various plants. Despite the proven effectiveness of elicitation techniques [104,105,106,107,108,109,110], research in this area on *E. longifolia* is insufficient.

#### 4.1.1. Biotic Elicitation

*E. longifolia* has been tested with only a few biotic elicitors, such as YE, JA, and SA. Many other biotic elicitors, including aspergillus and fusarium [111], remain unexplored. Additionally, effective elicitation agents like algae [112,113], proteins, and plant-growth-promoting microorganisms such as rhizobacteria and trichoderma [114,115], etc., have not been investigated. This represents a gap in academic research. 

#### 4.1.2. Abiotic Elicitation

Although physical elicitation methods are highly effective [115], limited research has been conducted on *E. longifolia*. While UV and gamma radiation have been tested, the details of their mode of action remain unclear. Furthermore, essential abiotic elicitation techniques such as LED [116] and nanoparticles [117] have not been explored. 

Different LED monochromatic lights have been employed individually or in combinations to enhance the synthesis of crucial metabolites. LEDs have the ability to improve metabolite production in both callus [118,119,120,121,122] and cell suspension [123,124]. Moreover, several studies highlight the effectiveness of LEDs in enhancing the synthesis of bioactive compounds in various in vitro cultures [116]. Additionally, numerous research papers document the efficacy of nanoparticle elicitation in enhancing metabolite production [125,126,127,128]. Therefore, there is a need to explore these aspects in *E. longifolia*.

### 4.2. Modern Breeding Strategies for Improved Biosynthesis

Modern molecular breeding techniques have been employed to enhance the synthesis of various plant substances. Initially, molecular markers [129] were used to identify existing variations in the population. Subsequently, omics technologies [130,131,132] can be utilized to identify the specific biosynthetic pathways and their underlying genes. In ginkgo, for instance, transcriptomics and metabolomics have been instrumental in improving bioactive metabolites [133,134,135,136]. Similar techniques have been applied to enhance the biosynthesis of taxol and other metabolites in yew trees [137,138,139,140,141]. Additional recent and efficient techniques include metabolic engineering and synthetic biology [142,143,144,145,146,147] for directly enhancing the synthesis of target metabolites.

The application of modern breeding technologies in *E. longifolia* is still in its early stages. Currently, only a limited number of studies have explored genetic variation in *E. longifolia*. These studies have utilized molecular markers such as inter-retrotransposon amplified polymorphism (IRAP), single-nucleotide polymorphism (SNP), random amplified polymorphic DNA (RAPD), simple sequence repeat (SSR), and microsatellites [148,149,150,151]. Additionally, DNA barcoding has been employed for the characterization of *E. longifolia* [152]. There is a clear need to expand research in this field to enhance the synthesis of bioactive metabolites, highlighting a substantial gap in the overall biotechnological exploration of *E. longifolia*.

## Figures and Tables

**Table 1:** Summary of the techniques for organogenesis in *E. longifolia* showing the explant and the media conditions used with the corresponding morphogenic response.

Explants	Media + PGR	Other Culture Conditions	Morphogenic Response/Outcome	Refs.
Shoot tips	BAP, KIN, and Zeatin	16L: 8D lighting of 150 µmol/m^2^/s	5.0 mg/L KIN induced shoots while 0.5 mg/L IBA induced roots	[51]
In vitro roots and stems	Shooting: MS, DKW or WPM + BAP, KIN, and Zeatin Rooting: MS + IBA	16L: 8D lighting of 150 µmol/m^2^/s	DKW + 1.0 mg/L kinetin + 1.0 mg/L zeatin formed shoots on root explants while WPM + 2.0 mg/L BAP + 2 mg/L zeatin formed shoots on stem explants MS + 0.5 mg/L IBA formed roots on shoot explants	[52]
Nodal segments	Shooting: BAP + KIN Rooting: IBA	Continuous light	½ MS + 0.5 mg/L BAP produced shoots while ½ MS + 10 mg/L IBA produced roots	[53]
Cotyledons	Shooting: BAP + KIN and TDZ Rooting: IBA + NAA	16L: 8D lighting of 35 µmol/m^2^/s	1.0 mg/L BAP and 0.5 mg/L IBA produced better shoots and roots, respectively	[54]
In vitro leaves	Shooting: MS + BAP; KIN and TDZ Rooting: ½ MS + IBA and NAA	16L: 8D lighting of 35 µmol/m^2^/s	1.0 mg/L BAP produced shoots directly; and ½ MS + 0.5 mg/L IBA produced roots on the shoots	[55]

Key: PGRs = plant growth regulators; BAP = 6-benzylaminopurine; KIN = kinetin; IBA = indole-3-butyric acid; TDZ = thidiazuron; NAA = 1-naphthaleneacetic acid; MS = Murashige and Skoog medium; DKW = Driver and Kuniyuki Walnut medium; WPM = woody plant medium.

**Table 3:** Techniques for the induction of somatic embryos (SEs) in *E. longifolia* showing the explant, media and culture conditions and morphogenic response.

Explants	Culture Media + Additives	Other Culture Conditions	Morphogenic Changes/Results	Refs.
Immature cotyledons	MS + 2,4-D, NAA, KIN, and BAP	Both light and darkness	Direct SEs produced on NAA while indirect on 2,4-D	[71]
All plant parts	MS + 2,4-D, IAA, IBA, dicamba, and NAA	16L: 8D lighting of 150 µmol/m^2^/s	Cotyledons on 1 mg/L 2,4-D produced embryonic callus (EC) and subculture of EC on 0.5 mg/L KIN, and 1 mg/L 2,4-D produced higher yield of SEs	[72]
Secondary callus	MS + 2,4-D and BAP or KIN	12L: 12D lighting and agitated at 120 rpm	1–2.5 mg/L 2,4-D, 2 mg/L BAP, and KIN produced SEs	[73]
Cotyledons	Modified MS + IBA, Zeatin, and TDZ	Activated charcoal and in dark	0.1 zeatin + 0.2 IBA + 0.12 TDZ produced highest SEs, and 2 mg/L IBA + 0.075 mg/L TDZ produced secondary SEs	[74]
Primary SEs	MS + IBA + Zeatin + TDZ and 0.1 g/L AC	RITA ^®^ bioreactors	Immersion rate of 5 min every 4 h produced highest number of SEs	[75]
Cotyledons	MS + 0.2 mg/L IBA + 0.1 mg/L Zeatinand 0.12 mg/L TDZ	Complete darkness	Globular SEs were produced	[75]

Key: SE = somatic embryo; EC = embryonic calli; BAP = 6-benzylaminopurine; KIN = kinetin; IBA = indole-3-butyric acid; TDZ = thidiazuron; NAA = 1-naphthaleneacetic acid; 2,4-D = 2,4-dichlorophenoxyacetic acid; MS = Murashige and Skoog medium.

**Table 6:** Techniques for *A*. *rhizogenes* transformation and/or induction of hairy roots (HRs) in *E. longifolia* showing different co-culture techniques and their outcome.

Starting Material	*A. rhizogenes* Strain and Media	Inoculation/Transformation	Initial Co-Culture Conditions	Subculture Media	Outcome	Refs.
Somatic embryos (SEs)	AR12 and AR14 strain grown in LB medium	SEs immersed in bacterial suspension for 20 min	Infected tissue inoculated in MS + 0.5 mg/L IBA and 1% PVP in dark	MS + 500 mg/L cefotaxime	Transient GUS expression observed in the explants	[95]
In vivo and in vitro plants, seedlings, embryos, and SEs	MAFF106590, 106591, 201265, 301726, and 720002 strains in LB medium	*A*. *rhizogene* suspension injected into explants	Infected tissues inoculated in MS in dark	MS + 300 mg/L cefotaxime	Only MAF201265, 301726 and 720002 induced HRs in hypocotyls region	[96]
Cotyledons and hypocotyl	ATTC 15834 strain in YMB medium	Explants incubated on bacterial plates for 30 min	Infected tissues inoculated on WPM under low light	WPM + 500 mg/L cefotaxime	HR tips appeared from the explants	[39]

Key: LB = Luria–Bertani media; YMB = yeast mannitol broth medium; WPM = woody plant medium; MS = Murashige and Skoog medium IBA = indole-3-butyric acid; PVP = polyvinylpyrrolidone; GUS = β-glucuronidase.

## Data Availability

No new data were created or analyzed in this study. Data sharing is not applicable to this article.
